# Tendencies and characteristics of public fitness behavior in the digital era: an Internet ethnography study based on the K Community in China

**DOI:** 10.3389/fpubh.2024.1414837

**Published:** 2024-10-21

**Authors:** Chen Jia, Songdong Ye

**Affiliations:** ^1^School of Physical Education, Guangdong University of Education, Guangzhou, China; ^2^School of Physical Education & Sports Science, South China Normal University, Guangzhou, China

**Keywords:** physical activity, fitness behaviors, digital technology, qualitative research, public health

## Abstract

**Introduction:**

The deep integration of media technologies into fitness activities in the era of digital intelligence has created a new and evolving landscape for public fitness. This transformation significantly impacts the practices and experiences of fitness.

**Methods:**

This study employs Internet ethnography as a qualitative research methodology, focusing on the daily activities of participants in a digital fitness community known as the K-community. Through immersive observation and analysis, the research seeks to uncover the evolving patterns and characteristics of fitness practices in China during the digital era.

**Results:**

As media technology and network culture advance, participants in digital fitness increasingly display fragmented, digitized, entertaining, ritualized, and highly socialized behaviors. These traits reflect the community’s desire for varied and complex exercise experiences within the context of modern technology.

**Discussion:**

The evolution of fitness behavior not only reflects the operational principles of contemporary media technologies but also encapsulates the essence of popular culture in the digital age. This study enhances our comprehension of fitness practices in digital environments and their wider cultural ramifications.

## 1 Introduction

Research has demonstrated that physical activity (PA) is a crucial factor in enhancing the health of individuals at all life stages (1). PA promotes normal growth, mental and physical health, sleep quality, and chronic disease prevention and reduction (2). Lack of PA is a confirmed risk factor for premature death and some non-communicable diseases (3). Despite the many benefits of PA for individuals and society, there are still 27.5% of adults (4) and over 81% of adolescents (5) who have not met the aerobic exercise recommendations proposed in the “2010 Global Recommendations on Physical Activity for Health” (6). Fitness apps have gained popularity due to COVID-19 restrictions, catering to public fitness and social interaction needs (7). The introduction of fitness apps has transformed the way people engage in fitness activities. Strategies such as setting explicit objectives, tracking progress, soliciting performance evaluations, accepting incentives, seeking social support, and gaining guidance from a coach appear to be particularly effective in promoting physical activity and fostering positive habits (8). Existing research mostly focuses on the following factors connected to fitness apps. First, research on the functional design of Fitness apps. Gratification is an important theoretical basis for the design of Fitness apps (9), for which the use of gamified and socialized design is beneficial to meet users’ needs for social interaction, entertainment, and enjoyment of Life (10). Existing fitness apps have functions such as GPS tracking, recording, motivation, and evaluation, which are beneficial for enhancing user motivation (11), accurately measuring energy intake and expenditure (12), precisely recording timely information on PA, and providing scientific evaluation (13). Second, regarding the functional preferences of Fitness apps and users’ attitudes and intentions. Fitness app success relies on a combination of functions, with motivational features attracting different user groups. Younger, female, high BMI users are more inclined toward gamified functions (14). Specifically, price, information quality, perceived entertainment value, perceived security risk, and perceived ease of use will affect users’ attitudes and intentions to use Fitness apps (15). Similar research has once again confirmed that users’ intentions to use Fitness apps are influenced by habits, perceived playability, health consciousness, perceived performance, and price value (16). Third, research has demonstrated that the implementation of five behavior change methods (BCTs) can significantly improve individuals’ physical fitness, cardiorespiratory fitness, and body composition. These strategies encompass routine self-monitoring, devising action plans and setting goals, evaluating behavioral objectives, providing performance feedback, and demonstrating the execution of desired behaviors within fitness applications. This is supported by other studies and a systematic review, confirming the effectiveness of fitness apps (17–19).

Fitness apps improve contemporary exercise habits by altering traditional fitness practices (11). Hence, it is imperative to investigate the patterns and attributes of public fitness behaviors within the framework of media technology. Unfortunately, prior research on public fitness behaviors in the mobile internet era has primarily relied on content analysis, randomized controlled trials, and cluster analysis, lacking the use of qualitative research to investigate novel characteristics (20). This paper aims to close the gap.

In the middle of the COVID-19 pandemic, China’s government regulations and the “Internet + Fitness” push have led to a significant increase in online fitness engagement. The number of users of fitness apps in China had reached 165 million by the end of 2019 (21). In 2020, the COVID-19 pandemic significantly boosted the popularity of digital fitness, resulting in a notable 93.3% surge in the number of people using digital fitness platforms (22). This study investigates the K Community, which is China’s largest user base for digital fitness, through the application of Internet ethnography and Meyrowitz’s media ecology theory. This study examines the impact of digital technology on public fitness practices, specifically inside virtual networks and digital environments. It provides valuable insights for advancing intelligent public fitness initiatives.

## 2 Methods

### 2.1 Exploring digital fitness practices: the selection of Internet ethnography methods

Internet ethnography is an ethnographic method that uses the internet’s virtual environment to study behavioral characteristics, interaction processes, and cultural meanings. It uses internet expression platforms and interactive tools to collect data and interpret cyberspace’s meanings (23, 24). Internet ethnography research involves various methods, typically including observation, participant observation, in-depth interviews, and text analysis. The operational steps mainly involve selecting and locating virtual fields, participant observation and data collection, and data analysis with reflective interpretation (25). This paper employs a relatively low-invasive internet ethnographic method to delve deeper into the current behavioral tendencies and characteristics of mass fitness in China. This method is based on observation and participant observation, supplemented by interviews and memos (26). It aims to examine the emerging fitness phenomenon from various perspectives, explore the impact of digital fitness practices on fitness users, and focus on the deeper meanings of mass fitness behavior in the digital age.

Based on the virtual field selection principles of relevance, frequency, heterogeneity, and richness, we established the K community as the observation field. The K Community, which is a pioneer in China’s exercise and fitness industry, has the most participants and active users. After gaining a preliminary understanding of the community through more than a month of pre-surveys, we immersed ourselves in the community for 9 months, gathering firsthand empirical data on the fitness participation of digital fitness users.

### 2.2 The virtual field presentation: in-depth immersion in the K fitness community

Launched in 2015, The K Community is a network fitness community that utilizes mobile internet technology. It offers a one-stop sports solution, including fitness instruction, socializing, dietary guidance, and equipment purchasing. With over 200 million registered users as of 2020, it is the hottest digital fitness platform in China (27).

After gaining a comprehensive understanding of the community’s access mechanisms, inherent attributes, section content, and humanized functions in July 2020, two researchers officially entered the K Community in August 2020. One of the two researchers is a user with 6 years of digital fitness experience (more insightful), and the other has never participated in digital fitness (more neutral). Both data collection and analysis adopt unified standards to ensure the objectivity of the data collection and analysis process to the greatest extent. The research journey, which included three stages of being an observer as a fitness participant, actively interacting as a fitness user, and reflecting as a researcher, lasted for 9 months. This study adopts purposive sampling. Initially, we entered the community for covert observation after explaining the research purpose to the community gatekeepers, collecting archival data such as posts (215), images (3304), emoji comments (1986), and audiovisual videos (122) from the popular sections of the K Community that reflect the behavior and attitudes of fitness participants (obtained by copying from the online community and members’ existing computer-mediated communication). Subsequently, we gradually identified ourselves, and, according to Delbert Miller, the usual practice in qualitative research is to interview 20–30 people (28). So, we sent online interview invitations to 38 interactive people, 26 active users, and 9 opinion leaders who have more than half a year’s fitness experience and posted and commented frequently in the K Community. In the end, 29 interactors (see Table 1), 17 active users, and 4 opinion leaders (see Table 2) accepted our online interview invitation. We questioned the participants about their daily fitness experiences and attitudes toward digital fitness. (1) What were the motivations behind your decision to engage in digital fitness? (2) What kind of experience have you gained from participating in digital fitness? (3) How has digital fitness changed you? (4) What are your thoughts on digital fitness? Finally, we collected nearly 150,000 words of field notes—obtained from written research reports, memos, and observation logs—through observation, recording, organizing, feedback, and reflection on the interactions and meanings of K Community members. These first-hand archived data, bootstrapped data, and field notes collected through internet ethnographic methods provide a more in-depth examination of user participation in digital fitness compared to quantitative research methods. As we gradually learn more about the real K Community and continue to enter and reinterpret it, we discover and comprehend the new elements contributing to mass fitness in digital experiences, mediated contexts, fluid spaces, and interactive arenas. The entire research process strictly followed ethical principles such as informed consent, compensation for interviewees, and privacy of participants. And we were approved by the Institutional Review Board of the corresponding authors.

**Table 1:** Selected online interactive users in the K Community.

Users	Activity levels	Fitness types	Users	Activity levels	Fitness types	Users	Activity levels	Fitness types
@L*	KG14	Running	@Y***	KG6	Aerobics	@J***	K15	Aerobics
@C***	KG15	Strength	@N****	KG11	HIIT	@X***	KG16	HIIT
@O**	KG12	HIIT	@X***	KG10	HIIT	@S**	KG12	Running
@P**	KG19	HIIT	@C***	KG17	Running	@D**	KG17	HIIT
@X***	KG7	HIIT	@D***	KG9	Aerobics	@D***	KG13	HIIT
@J**	KG5	Yoga	@A**	KG11	Yoga	@Q***	KG8	Aerobics
@A**	KG9	Yoga	@D**	KG9	Yoga	@J***	KG9	Strength
@L***	KG11	Strength	@W*	KG6	Aerobics	@W**	KG12	Running
@F**	KG12	HIIT	@L***	K14	HIIT	@M***	KG9	Yoga
@R**	KG7	Running	@C***	KG11	HIIT			

To protect user privacy, **** is used to hide their online usernames. KG: In the K Community, a higher KG value denotes a higher frequency, duration, and activity level of fitness participation.

**Table 2:** Online interview participants selected from Community K.

Interviewee	Gender	Age	Duration	Type	Interviewee	Gender	Age	Duration	Type
P1	F	29	7 Month	Yoga	P12	F	38	30 Month	Aerobics
P2	F	34	14 Month	Yoga	P13	M	29	18 Month	HIIT
P3	F	23	24 Month	HIIT	P14	M	31	21 Month	Mixed aerobics
P4	F	27	18 Month	HIIT	P15	M	28	24 Month	HIIT
P5	F	33	49 Month	Strength	P16	M	26	20 Month	HIIT
P6	F	25	6 Month	Running	P17	M	23	6 Month	HIIT
P7	F	30	20 Month	Yoga	P18	M	41	84 Month	Strength
P8	F	32	30 Month	HIIT	P19	F	26	60 Month	HIIT
P9	F	30	7 Month	Yoga	P20	F	47	32 Month	Running
P10	F	36	63 Month	HIIT	P21	F	23	36 Month	Strength
P11	F	24	6 Month	Strength					

## 3 Results

Emerging mobile fitness media technologies have transformed traditional fitness experiences by embedding behaviors within virtual networks and digital landscapes, transforming fitness behaviors from physical spaces to virtual networks and digital landscapes (29). The study on Community K reveals that in the digital age, fitness users’ exercise participation behaviors have evolved due to technological advancements and cultural shifts, exhibiting trends of fragmentation, digitization, entertainment, ritualization, and socialization.

### 3.1 Fragmentation: examining the fragmentation and fluidity of fitness in time and space

In the current era where 5G technology, smartphones, and digital terminals permeate everyday life practices, the emergence and popularity of new fitness modalities based on the internet and new media are changing the storage forms of fitness information and the ways of acquiring bodily experiences. This has resulted in the division of fitness behaviors among exercise participants, which is marked by a distinct separation of time and flexibility of space. On the one hand, time, as one of the fundamental material dimensions of human life, is not only a basic intrinsic variable of social development but also an important measure of social behavior (30). Fitness in the digital age, with its portable and instant media characteristics, breaks the temporal limitations and oppression of past exercise participation. In addition to providing the convenience of engaging in physical activities at any time, platforms like K offer various fitness courses such as “3 min Swan Neck Training,” “5 min Explosive Manoeuvre,” and “One Song Fat Burning Exercise” of different durations, intensities, functions, and types. These courses repurpose the previously “idle” in-between time—brief, temporary, fragmentary, and practically meaningless moments (31). The majority of users in the K Community reported: “Cycling with a fitness app on the way home from work” (@J***, K15), “Practicing a set of shoulder and neck relaxation yoga during lunch break” (@A**, K11), and *“Doing some strength training with videos during the children’s post-dinner free time” (P12, 38 years old)*. Modern society has seen an increase in multi-segment, short-duration, high-frequency exercise patterns as a result of the accelerated pace of work, the consumption of personal leisure time by various socialized identities, and urbanization. Individuals in the mobile internet era desire to participate in exercise while dealing with different social lives, resulting in time fragmentation to meet exercise needs, commute issues, physical and mental health, and professional development goals. This training setting matches the “digital existence” needs of today’s people.

On the other hand, the spatiotemporal structure of the network society is undergoing a transformation from traditional local space to fluid space, also known as the space of flows (30). With the superior mobility and portability of mobile smart devices, digital fitness in the mobile internet era has dissolved the boundaries between physical and digital spaces, liberating the body from fixed geographical locations (32). People’s current exercise behaviors are more “fragmented” compared to past exercise behaviors in designated places and at dedicated times. The majority of users in Community K (Participant 14, 31 years old), *“It’s precisely because media technology can extend the fitness field to cover homes, workplaces, communities, outdoors, park squares, gyms, and even the roads leading to them (in-between space).”* This means that the public’s participation in fitness activities or professional training is no longer limited to specific spaces. The vigorous development of this more open and activity-based rather than location-based fitness approach not only broadens the flexibility of individual exercise space choices and strengthens control over personal exercise spaces but also enhances the diversity of daily workouts for fitness participants. Furthermore, the combined influence of work hours, commuting patterns, and residential locations (33), as well as gender responsibilities and household division of labor, intensifies the spatial fragmentation of public fitness behaviors (34, 35).

### 3.2 Digitalization: body presentation visualization and data-driven operation

With the decreasing costs of wearable devices, sensor technology, mobile smart terminals, and the increasing ubiquity of fitness media, fitness activities based on information technology are becoming a new way of life and interaction (36), gradually showing a tendency toward “datafication.” This datafication trend is evident in how fitness participants display their fitness trajectories, how they present their physical status, and how they showcase their exercise achievements.

**Figure 1:**
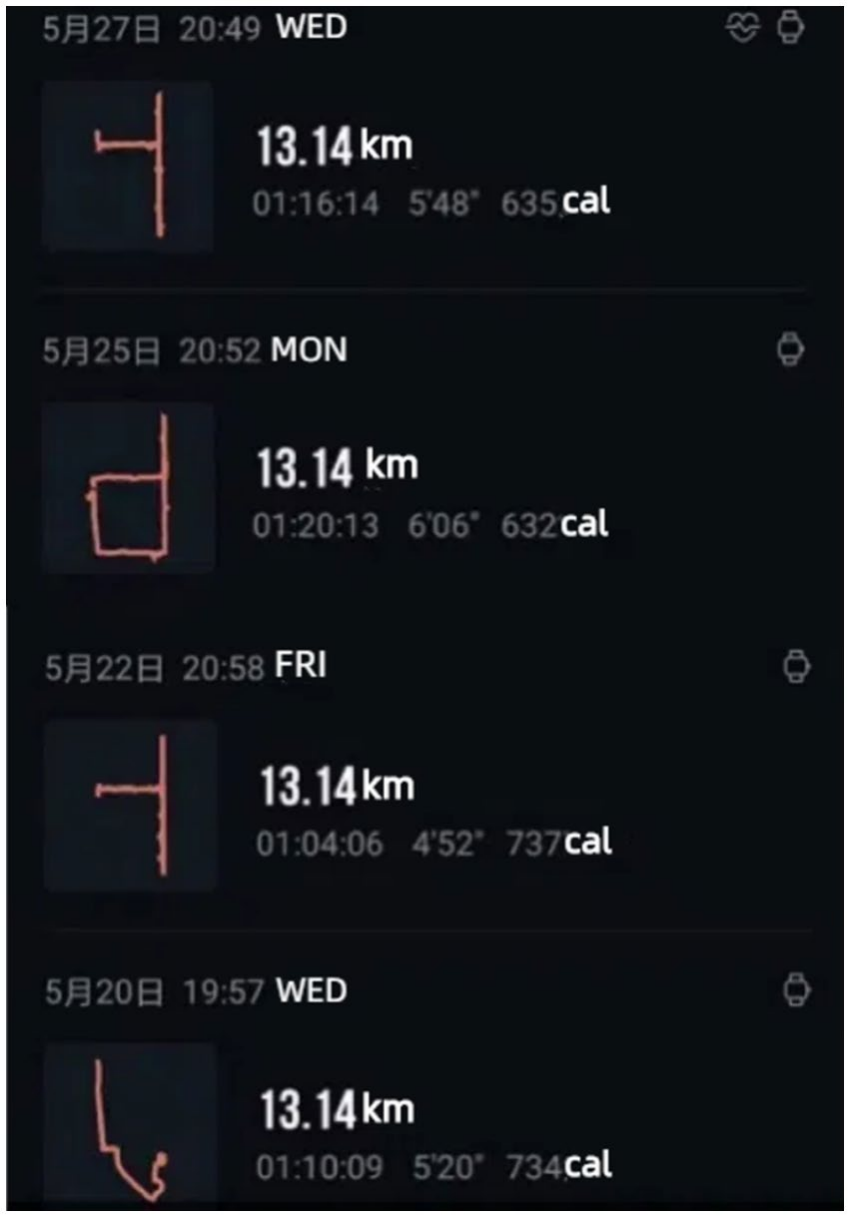
Digital running records.

**Figure 2:**
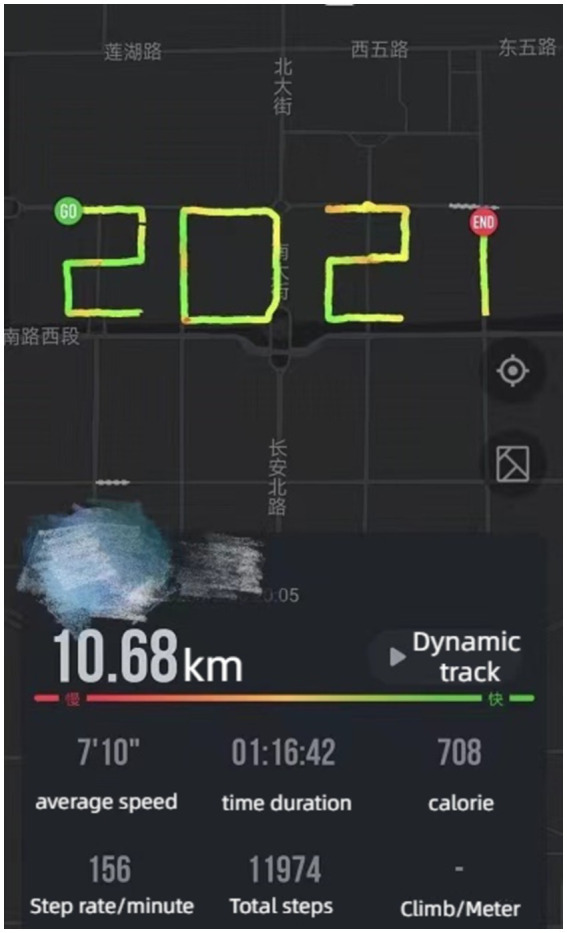
Digital running path.

First, as digital data recording and storing of human behavioral traces has grown in popularity and demand in recent years (36, 37), media technology has given previously boring and monotonous exercise data more fun and extensibility, enabling fitness participants to assert, display, and review themselves. By analyzing the observations of the K Community, it is evident that fitness participants in the current era of mobile internet not only use GPS positioning to track their location, but also document their fitness journey, lifestyle, and outlook on life through weekly or yearly reports that feature visual evidence of their progress and initial starting point. In addition, they employ their bodies as “brushes” to artistically document personal experiences through the use of fitness media and the monitoring capabilities depicted in Figures 1, 2 (@X***, K16), showcasing their distinctiveness and artistic perspectives. Therefore, by incorporating highly accurate numerical data, the digitized fitness footprints provide added value that extends beyond fitness.

Furthermore, with the advent of the data-driven era of the quantified self, the functional types of fitness media have gradually expanded from tracking physiological indicators to monitoring body shape data. This shift has encouraged an increasing number of fitness participants to participate in fitness practices that manage and express their physical state in a data-driven manner. One can regularly upload their body data to the cloud, including height, weight, and body dimensions, to create intuitive body shape graphs (@X***, KG10). They can also record physiological indicators during exercise, such as calorie consumption, heart rate, blood pressure, body fat, and lung capacity, or even showcase their external body through digital imagery (@W**, KG12). The popularity of digital fitness has enabled people to transition from a subjective experience to an objective, visual consideration of their physical state in their daily fitness practices (38), especially under the “cultivation” of convenient features such as automatic monitoring and proactive reminders from fitness applications. Pursuing a digital body shape has become a lifestyle belief for fitness participants. These seemingly “real,” “credible,” and “scientific” visualized body data have propelled the trends of “quantified self” and “body digitalism”.

In the era of mobile connectivity, where fitness participants can experience every aspect of daily life in a quantified way through mediated technology (39), the journey to document every workout and celebrate every compliment and milestone has begun—a digital voyage showcasing individual athletic achievements (likes, followers, comments, exercise levels, and rankings). For the fitness community within the K platform, the likes, attention, and volume of comments garnered during exercise represent more than just tech-driven social interactions; they signify recognition of individual fitness behaviors, attitudes, identities, and values. In the digital fitness process, visualized data empowers fitness participants to assert, “You are your data” (40). As media technology surpasses the constraints of subjective emotions, it affirms self-achievement in a more objective manner (41). In today’s internet age, where exercise experiences and fitness results are no longer mere personal recollections but observable, tangible, and shareable digital events, digitizing fitness participation has become increasingly prominent.

### 3.3 Entertainment: the fun and gamification of exercise participation

The advent of the internet and new media has revolutionized the concept of exercise, making it a more engaging and enjoyable activity. Exercises now incorporate various entertainment features and gaming elements that enhance physical fitness. This has resulted in a more gamified and enjoyable approach to exercise and fitness interactions, with training becoming a monotonous and repetitive activity.

**Figure 3:**
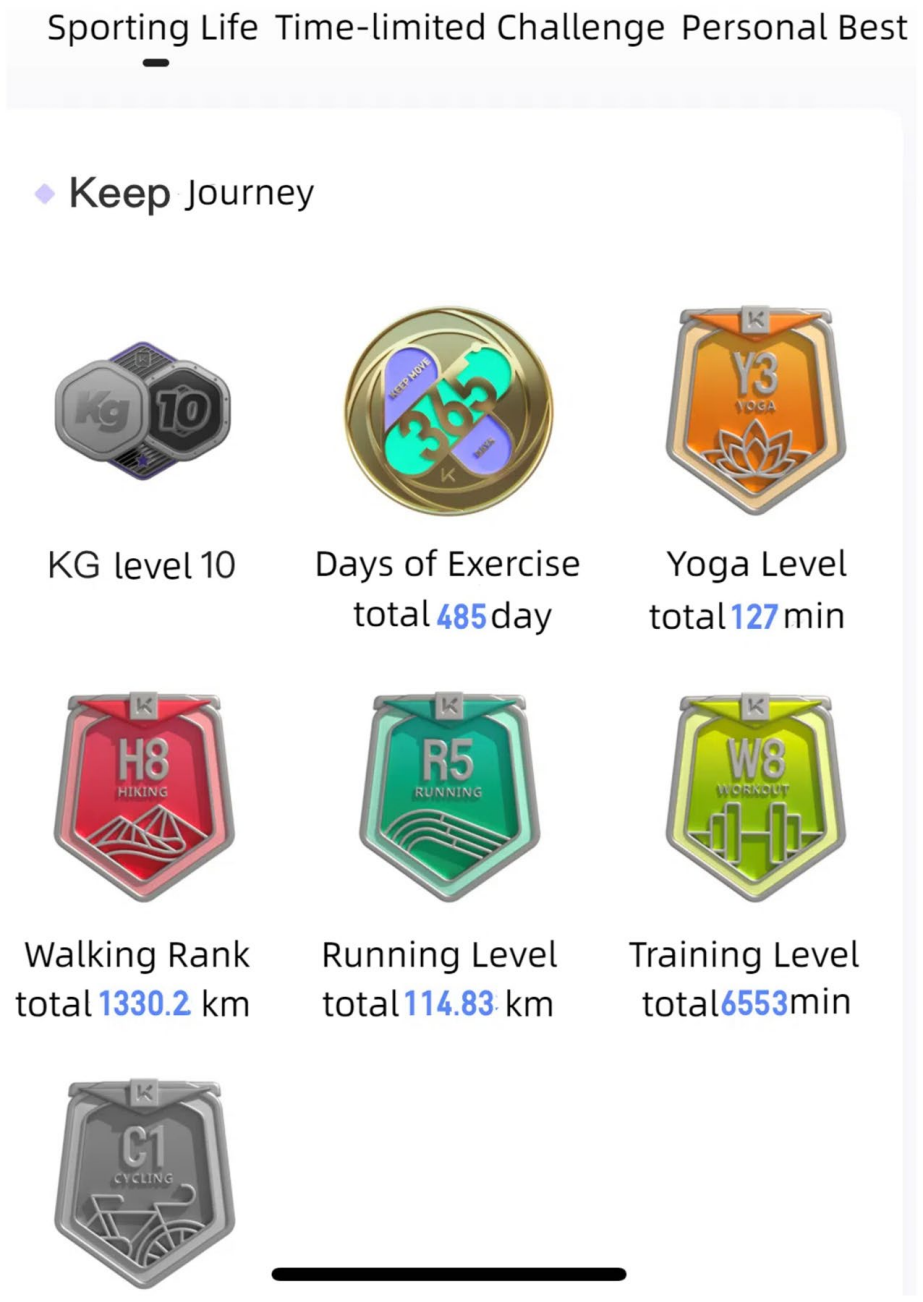
Digital fitness career.

**Figure 4:**
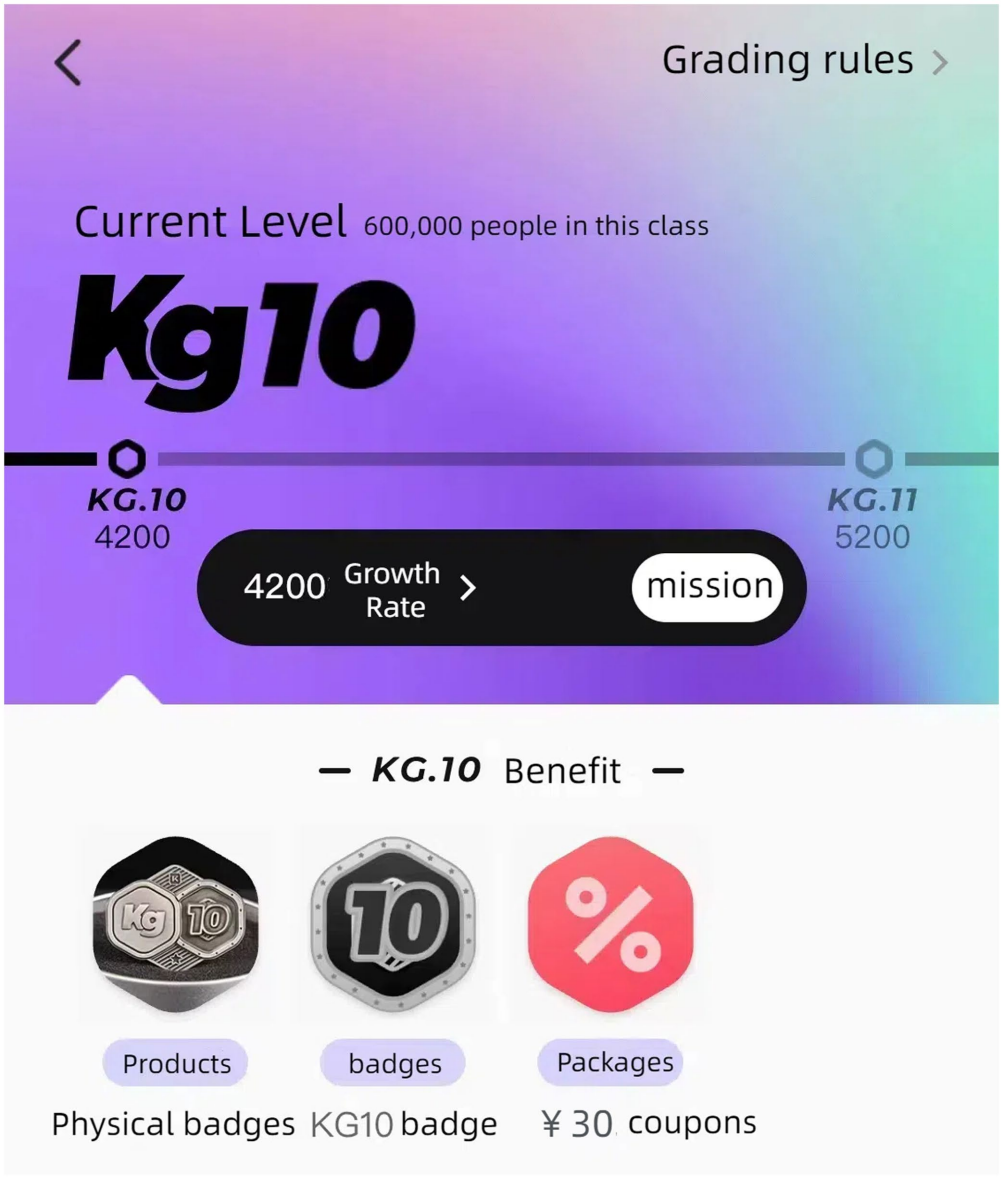
Digital fitness level.

The K Fitness Community aims to transform the public’s impression of fitness activities as boring and monotonous. It achieves this by combining the inherent processes of fitness activities with digital games, resulting in increased user engagement and enjoyment. The K Fitness Community enables the “Born Digital” generation, who are accustomed to a digital world, to participate in more engaging fitness activities by incorporating various elements of play and exercise. These include earning points by tracking mileage, collecting medals through exercise, and receiving rewards for check-ins, as shown in Figures 3, 4 (42). These individuals, who were born in the 80s and 90s and are a key part of the fitness community, are highly adaptable and open to incorporating trendy and intriguing gaming elements into their fitness routines. As a result, they are more likely to be drawn to the virtual gaming scenarios created by digital fitness media. Media technology utilizes multidimensional game features, rich motivational approaches, and engaging experiences to generate a process known as “happy activities.” This process, in turn, fosters an attitude to exercise participation that is centered upon enjoyment (43).

On the other hand, the proliferation of mobile intelligent terminals and media technology in the digital age provides a foundation for embedding various game elements or mechanisms into human daily fitness practices. The popularity of features and content, such as the fitness friend’s leaderboard and exercise task challenges (player killing) in Community K, intensifies the fitness participants’ perception of competition and victory as the essence of exercise. “Competition and winning make sports,” said P18, a 41-year-old, expressing their desire for real-time visibility of their exercise ranking. “It’s exciting to let my fitness friends see my workout rank in real-time” (@L***, KG11). The popularity of fitness ranking stems from its ability to appeal to individuals’ competitive and demanding inclinations, facilitate comparison and contact among people, and meet their desire for active involvement, presence, and self-identification. As a result, it encourages fitness participants to increase their engagement and physical experience in fitness—a non-gaming context. Whether it’s the exercise leaderboard, a representative of indirect competition in gamification (44), or the exercise PK match, a representative of direct competition in gamification (45), both are significant sources of motivation that evoke a sense of participation, excitement, achievement, and connection in the exercise context. The technological characteristics and media logic of online fitness platforms in the mobile internet era gradually shape the gamified interaction behaviors of fitness participants, embodying a positive approach to interpersonal interaction and social relationship construction.

### 3.4 Ritualization: the fitness journey’s representation and performance

In today’s technological and media era, the importance of fitness for the general public has evolved beyond its practical purpose. This is largely due to media technology’s influence on portraying health ideals and shaping body perceptions. As a result, fitness has started to encompass more socio-cultural dimensions. When individuals engage in fitness activities with the intention of communicating or performing (46), their actions, such as setting goals before entering the gym, giving feedback during workouts, and sharing post-workout images, naturally take on ritualistic qualities.

Firstly, due to the increasing popularity of social media in recent years, it has been common practice to establish weight reduction goals. The era of mobile internet has rearranged and reorganized conventional norms and culture, such as “military orders” and “wishing,” in a novel manner. Historically, military orders and well-wishing practices typically entailed the formal signature of a mission promise prior to engaging in combat, accompanied by the expression of favorable desires. In the era of mobile internet, traditional rituals are becoming less complex and less sacred. However, the use of textual and visual symbolic metaphors helps to maintain the spirit and emotional impact of the weight loss flag ceremony. Thus, within the K Community, those who are new to fitness typically establish ambitious objectives with a competitive mindset, such as *“Either achieve weight loss or face dire consequences!”* In order to motivate themselves, individuals (@X***, KG7) actively seek inspiration. The approach to establishing fitness objectives, which involves a combination of self-deprecating humor and sincere supplication, is indicative of the anxiety individuals experience regarding their physical appearance due to the societal emphasis on the ideal body image in digital consumer culture. Furthermore, it serves as a medium for individuals to convey their unique sense of self and principles, which they actively seek to acquire in order to achieve emotional solace and spiritual reinforcement. In the era of digital fitness, individuals participating in exercise are unconsciously transferring old rituals onto online platforms in an effort to delegate their weight loss aspirations to external entities by establishing fitness objectives (47).

Second, exercise check-ins, which are part of the “Internet + Sports” application in nationwide fitness, have become a common activity pattern in recent years (48). These check-ins also serve as a behavior for recording and displaying to achieve recognition and resonance (49). As a form of media practice, fitness participants, whether they are passively completing the sign-in tasks set by the system after finishing their exercise plans or actively providing feedback on their workout conditions and results after participating in exercise, are becoming a widespread and frequent ritualistic behavior in the fitness process during the mobile internet era. Individuals or specific groups engage in this check-in behavior in daily life, both in expression and practice (50), which is more akin to the long-standing tradition of “keeping a diary” in Chinese history and represents a new manifestation of the diary-writing ritual in the mobile internet age. The check-in reminder function in the K Community, as well as the standardized and proceduralized process settings, provide a media mirror for fitness participants to reflect their self-planning awareness, personal quality traits, and sense of order in daily life. Fitness participants will become more consciously invested in the daily practice of exercise check-ins as this perception of personal identity recognition and self-presentation amplifies in online communities (51), transforming the recording of oneself into a stable and continuous everyday media ritual.

Finally, digital technology has given rise to a new fitness media environment. As fitness participants acquire “megaphones for expression and cameras for dissemination,” they also carve out a new realm for self-fashioning and identity affirmation. Thus, “fitness sharing” emerges as a distinct cultural form (52). As a result, “fitness sharing” emerges as a distinct cultural form. A seven-month immersive investigation into the K Community reveals that the act of “sharing” within this spectacularized fitness media environment, steeped in digital technology and image consumption culture, is no longer an occasional, singular event but has become the predominant lifestyle of the fitness demographic. *“From sharing workout data to fitness selfies, from flaunting sportswear to fitness equipment, from showcasing fitness meals to sharing exercise insights” (P4, 27 years old)*, “sharing” has indeed become a “national sport” in the fitness circles of the mobile internet era, increasingly evolving into a public performance ritual (53, 54). For fitness participants, “sharing” is not only a mode of self-disclosure that carries individual emotional appeals, value identification, and the quest for belonging, but also a form of role-playing and selective presentation in a mediated social environment. Carefully planned self-performances and the interaction of others’ recognition gradually push and draw fitness participants into “living for sharing” fitness practices.

### 3.5 Socialization: stratification and interaction in relational connections

Digital fitness has transformed into a platform for emotional interaction and social connection, with features like sports leaderboards, fitness circles, and local meetups. This interactive nature has accelerated communication within fitness communities, promoting exercise socialization. The “no socialization, no exercise” mantra reflects the fitness community’s individuality and external behavior (55).

In a network society characterized by fragmented time, fluid space, and interpersonal alienation, social interaction has gradually become a rigid necessity for people, particularly after media intervention in the fitness domain. Fitness participants engage in a novel interaction mode of “absent bodies but present attention” through digital symbols transformed and generated by media technologies such as text, images, and emojis, as well as built-in technologies like sensors and GPS positioning. Leveraging media technology, fitness participants can interact and communicate through continuous switches between simulated environments and real-life scenarios. This has prompted an increasing number of users to move beyond self-admiration in exercise and join the ranks of those sharing their fitness journeys. In the K Community, the interactive act of “sharing” is not only one of the motivations to participate in exercise but also an important means to establish, maintain, and expand relationships. Since media technology places fitness participants in simulated exercise reflections, the mediatization weakens the sense of presence and intimacy in real life. Fitness participants urgently need to demonstrate “I am with everyone” through actions like “following,” “liking,” and “commenting,” in order to satisfy their social and emotional needs and gain attention (56). It is precisely under the weakening network structure of social interaction and interpersonal relationships in the mobile internet era that people pin their hopes on online interactions to reconstruct new social relationships, thus forming an exercise interaction model centered on “sharing” and aimed at expanding one’s social life.

Conversely, as digital technology continues to expand and integrate into everyday life, the concept of a communal “digital existence” has become the new standard, and individuals are becoming more accustomed to joining online communities. In the digital fitness realm, the rise of fitness media has led to the emergence of jogging clubs, exercise check-in groups, and fitness equipment mutual aid circles centered around communal fitness activities. These have steadily gained popularity and become a trend. In the K Community, “whether it’s the periodic online marathon running events within the group” (@L*, KG14), *“the regular exercise check-in relays in the chat group” (P9, 30 years old), or “the seeding and venting about fitness equipment within the circle” (P21, 23 years old)*, the homogenized value orientation and exchange of information resources are key to the formation and maintenance of the exercise interest community. When people with common hobbies, similar exercise experiences, and emotional experiences establish a strong sense of belonging and stable dynamic structures within a “sameness” and “tribal” social atmosphere, and when emotionally connected individuals can enhance internal collaboration and resource exchange through mutual understanding and recognition, these driving forces, such as huddling for warmth, mutual support, and resource exchange, naturally encourage a significant number of fitness participants to join the trend of “adding circles” and “grouping,” ensuring the stable development of their relationship networks through shared beliefs and mutual benefits.

## 4 Discussion

### 4.1 Technological empowerment in the realm of online fitness

With the rapid development of the internet and new media, the emergence of new media technology functions such as online teaching, live streaming, instant interaction, data recording, and information dissemination not only empowers individuals but also profoundly reshapes the public’s fitness behaviors.

On the one hand, media technology is revolutionizing fitness by introducing various exercise participation modes, breaking traditional fitness’s spatial and temporal limitations. This empowers the fitness community, allowing them more autonomy and flexibility. Digital fitness content, combining visibility and gamification, enhances public fitness behaviors by promoting autonomous exercise habits and increasing scientific exercise awareness. It also reactivates people’s sense of community and attention to exercise in diverse scenarios, pushing public fitness toward socialization. These behavioral changes reflect the basic logic of media technology, which facilitates scientific, convenient, diversified, immersive, and interactive participation in national fitness.

On the other hand, the mobile, flexible, and autonomous fitness forms generated by integrating the internet and media technology into the fitness domain physically fragment participants, turning them into atomized individual entities. The loss of fixed and concrete spatial environments characteristic of traditional exercise disrupts the stability and continuity of exercise time, leading to a scattered, fragmented, and unstructured order of physical activities. Furthermore, in the era of mobile internet, the “digital body,” measured and represented by data, exacerbates data misuse. This could potentially shift the trend of smart growth in national fitness toward the alienation of body performance, as “body digitalism” increasingly infiltrates the private sphere (54). Hence, in the era of “digital existence,” as we logically acknowledge the subjective sensations of the body facilitated by technical advancements, we must also calmly confront the chaotic progress and privacy breaches caused by technology. Therefore, we may enhance the consistent development of intelligent national fitness in China by employing digital technology to connect the gaps between supply and demand and applying media technology to address the gaps in information and knowledge.

### 4.2 Cultural evolution in the digital fitness

Images and data enhance communication and foster relationship development in a culture characterized by interconnected networks. The digital fitness landscape consists of symbolic media mirrors that possess intricate cultural significance. This landscape further divides individuals’ fitness habits as the influences of consumer culture, body culture, and popular culture intersect. The digital mirror image combines contemporary Western fitness principles with traditional Chinese cultural ideas, consumerism, aesthetics, and internet culture, resulting in the manipulation and deterioration of the body. They incorporate the concepts of regular exercise, scientific body evaluation, and healthy exercise lifestyles into people’s daily fitness routines. Additionally, they assign various cultural meanings and value labels to the physical forms of fitness participants, such as identity, taste, and capital. As a result, those participating in fitness activities, shaped by the concept of “body transformation,” begin to view the body as a tool for “efficiency” in the creation of the digital fitness environment (57). They consistently enhance the performative and representational features of their physical actions through a two-way interplay between accommodation culture and production culture. This process connects individual cultural capital, bodily capital, identity consciousness, and popular culture.

On the other hand, fitness participants are embracing the “body transformation” value system, viewing the body as a productivity tool in the digital fitness industry. They improve the expressive and symbolic elements of their physical actions by incorporating their personal cultural knowledge, physical abilities, self-awareness, and mainstream culture. However, due to excessive immersion in the digital landscape and internet culture, fitness participants must focus on preserving social beliefs and group emotions. As a result, fitness activities in the mobile internet era lose their authentic essence and exhibit distinct stratification and segmentation characteristics. It is critical to be cautious of the isolation that can occur in public fitness behaviors as a result of changes in network structure and society’s cultural evolution. It is critical to promote intelligent national fitness growth by focusing on exercise interconnection, identity co-construction, and cultural integration.

## 5 Conclusion

This study explores the transformation of exercise behaviors among fitness participants in China during the digital age. Through an immersive investigation of the K Fitness Community, we find that the fitness participation behaviors of Chinese fitness users have undergone reconstruction and reformation in the context of technological empowerment within virtual fitness environments and cultural shifts in digital fitness landscapes. These behaviors exhibit notable tendencies toward fragmentation, digitization, entertainment, ritualization, and socialization. The scattered nature of users’ workout times and the fluidity of fitness spaces primarily reflect the fragmentation trend. The digitization trend manifests in the data representation of fitness trajectories, physical conditions, and exercise achievements. The entertainment orientation showcases the enjoyable aspect of user participation in exercise and the gamification of fitness interactions. The ritualization shift includes users establishing self-rituals around weight loss goals, daily rituals of exercise check-ins and fitness feedback, as well as performance rituals like showcasing workouts and self-promotion. Lastly, the socialization trend is characterized by user interactions centered on sharing and the formation of fitness networks based on shared interests.

## 6 Limitations

As mentioned above, despite the large number of digital fitness users, the user demographic typically comprises digitally literate young and middle-aged individuals. This study focused on interviewing participants primarily from this age group, leaving out older adults and minors; therefore, the findings may not fully represent the perspectives of the entire population across different age ranges regarding digital fitness. Furthermore, we conducted all interviews online, with participants typically possessing higher levels of digital fitness literacy. As a result, their insights may differ from those of users with lower levels of digital fitness literacy. Despite these limitations, digital fitness offers unique bodily experiences and emotional connections that differ from traditional fitness practices, leading to the formation of distinctive fitness behaviors. At the same time, the iteration of digital fitness technologies brings risks such as privacy exposure and the potential for technology to exert control over users’ bodies. Thus, it is important to approach the opportunities and challenges presented by digital fitness with a balanced perspective. Future research could focus on groups that have opted out of using digital fitness technologies.

## Data Availability

The raw data supporting the conclusions of this article will be made available by the authors, without undue reservation.

## References

[1] Ding D Mutrie N Bauman A Pratt M Hallal PR Powell KE. Physical activity guidelines 2020: comprehensive and inclusive recommendations to activate populations. Lancet. (2020) 396:1780–2. doi: 10.1016/S0140-6736(20)32229-7, PMID: 33248019

[2] Piercy KL Troiano RP Ballard RM Carlson SA Fulton JE Galuska DA. The physical activity guidelines for Americans. JAMA. (2018) 320:2020–8. doi: 10.1001/jama.2018.14854, PMID: 30418471 PMC9582631

[3] Katzmarzyk PT Friedenreich C Shiroma EJ Lee I-M. Physical inactivity and non-communicable disease burden in low-income, middle-income and high-income countries. Br J Sports Med. (2022) 56:101–6. doi: 10.1136/bjsports-2020-103640, PMID: 33782046 PMC8478970

[4] Guthold R Stevens GA Riley LM Bull FC. Worldwide trends in insufficient physical activity from 2001 to 2016: a pooled analysis of 358 population-based surveys with 1· 9 million participants. Lancet Glob Health. (2018) 6:e1077–86. doi: 10.1016/S2214-109X(18)30357-7, PMID: 30193830

[5] Guthold R Stevens GA Riley LM Bull FC. Global trends in insufficient physical activity among adolescents: a pooled analysis of 298 population-based surveys with 1· 6 million participants. Lancet Child Adolesc Health. (2020) 4:23–35. doi: 10.1016/S2352-4642(19)30323-2, PMID: 31761562 PMC6919336

[6] World Health Organization. Global recommendations on physical activity for health. Geneva: World Health Organization (2010). 26180873

[7] Zheng EL. Interpreting fitness: self-tracking with fitness apps through a postphenomenology lens. AI & Soc. (2023) 38:2255–66. doi: 10.1007/s00146-021-01146-8, PMID: 33584017 PMC7868075

[8] Sullivan AN Lachman ME. Behavior change with fitness technology in sedentary adults: a review of the evidence for increasing physical activity. Front Public Health. (2017) 4:289. doi: 10.3389/fpubh.2016.00289 28123997 PMC5225122

[9] Huang J Zhou L. Timing of web personalization in mobile shopping: a perspective from uses and gratifications theory. Comput Hum Behav. (2018) 88:103–13. doi: 10.1016/j.chb.2018.06.035

[10] Lim SL Bentley PJ Kanakam N Ishikawa F Honiden S. Investigating country differences in mobile app user behavior and challenges for software engineering. IEEE Trans Softw Eng. (2015) 41:40–64. doi: 10.1109/TSE.2014.2360674

[11] Molina MD Sundar SS. Can mobile apps motivate fitness tracking? A study of technological affordances and workout behaviors. Health Commun. (2018) 35:65–74. doi: 10.1080/10410236.2018.1536961, PMID: 30358424

[12] Yap SS Suparjoh S. Development of Mobile application for health and fitness guidance-FIT DAY. Appl Inf Technol Comput Sci. (2022) 3:217–37.

[13] Kranz M Möller A Hammerla N Diewald S Plötz T Olivier P. The mobile fitness coach: towards individualized skill assessment using personalized mobile devices. Pervasive Mob Comput. (2013) 9:203–15. doi: 10.1016/j.pmcj.2012.06.002

[14] Wang Y Collins WB. Systematic evaluation of mobile fitness apps: apps as the tutor, recorder, game companion, and cheerleader. Telematics Inform. (2021) 59:101552. doi: 10.1016/j.tele.2020.101552

[15] Erasmus L Venter De Villiers M Phiri N. Mobile app characteristics that influence usage intention of health and fitness apps among millennial consumers. J New Gen Sci. (2018) 16:40–61.

[16] Damberg S. Predicting future use intention of fitness apps among fitness app users in the United Kingdom: the role of health consciousness. Int J Sports Mark Spons. (2022) 23:369–84. doi: 10.1108/IJSMS-01-2021-0013

[17] Muntaner-Mas A Sanchez-Azanza VA Ortega FB Vidal-Conti J Borràs PA Cantallops J. The effects of a physical activity intervention based on a fatness and fitness smartphone app for University students. Health Informatics J. (2021) 27:1460458220987275. doi: 10.1177/1460458220987275, PMID: 33446036

[18] Berglind D Yacaman-Mendez D Lavebratt C Forsell Y. The effect of smartphone apps versus supervised exercise on physical activity, cardiorespiratory fitness, and body composition among individuals with mild-to-moderate mobility disability: randomized controlled trial. JMIR Mhealth Uhealth. (2020) 8:e14615. doi: 10.2196/14615, PMID: 32014846 PMC7055745

[19] Schoeppe S Alley S Van Lippevelde W Bray NA Williams SL Duncan MJ. Efficacy of interventions that use apps to improve diet, physical activity and sedentary behaviour: a systematic review. Int J Behav Nutr Phys Act. (2016) 13:1–26. doi: 10.1186/s12966-016-0454-y 27927218 PMC5142356

[20] Peng W Kanthawala S Yuan S Hussain SA. A qualitative study of user perceptions of mobile health apps. BMC Public Health. (2016) 16:1158–11. doi: 10.1186/s12889-016-3808-0, PMID: 27842533 PMC5109835

[21] Data YG. Comprehensive analysis of China’s online sports market in 2019. Beijing: Yi Guan Qian Fan (2019).

[22] Insight Report on the Impact and Implications of the COVID-19 Pandemic on Life in 2020. Available at: https://www.questmobile.com.cn/research/report-new/87 (Accessed June 26, 2023).

[23] Hine C. “Overview: Virtual Ethnography: Modes, Varieties, Affordances.” In: Nigel G Lee RM Blank G (editors). Handbook of Online Research Methods (2008) London: Sage, 257–70.

[24] Bu Y. Virtual ethnography: the field, approach and ethics. Soc Stud. (2012) 6:217–46. doi: 10.19934/j.cnki.shxyj.2012.06.010

[25] Duan Y. Network ethnography:how to explore the meaning production and cultural construction of online communities. Qinghai J Ethnol. (2019) 30:76–86. doi: 10.15899/j.cnki.1005-5681.2019.01.012

[26] Jia C Guo Q. Value response, applicability and reflection of internet ethnography asa research method in sports studies. J Wuhan Sports Univ. (2023) 57:25–32. doi: 10.15930/j.cnki.wtxb.2023.08.003

[27] Nielsen. Public fitness behavior and consumption research report, vol. 2020. Shanghai: China Sporting Goods Federation (2021).

[28] Miller DC Salkind NJ. Handbook of research design and social measurement. Thousand Oaks, CA: Sage (2002).

[29] Chen R. Local reconstruction:cultural metaphor of rural Youth's media behavior. China Youth Stud. (2019) 2:94–101. doi: 10.19633/j.cnki.11-2579/d.2019.0049

[30] Oliván MC. The rise of the network society. Beijing: Beijing Social Sciences Academic Press (2003).

[31] Ji N. The reshaping of time and space in Mobile communication: a review of foreign scholars’ research on the relationship between Mobile phones and time-space since 2000. Lit Art Stud. (2008) 12:62–72.

[32] De Souza e Silva A. From cyber to hybrid. Space Cult. (2006) 9:261–78. doi: 10.1177/1206331206289022

[33] Hubers C Schwanen T Dijst M. ICT and temporal fragmentation of activities: an analytical framework and initial empirical findings. Tijdschr Econ Soc Geogr. (2008) 99:528–46. doi: 10.1111/j.1467-9663.2008.00490.x

[34] Townsend AM. Life in the real-time city: Mobile telephones and urban metabolism. J Urban Technol. (2000) 7:85–104. doi: 10.1080/713684114

[35] Kwan M-P. Gender, the home‐work link, and space‐time patterns of nonemployment activities*. Econ Geogr. (1999) 75:370–94. doi: 10.1111/j.1944-8287.1999.tb00126.x

[36] Negroponte N. Being digital. New York, NY, USA: Vintage (2015).

[37] Freelon D. On the interpretation of digital trace data in communication and social computing research. J Broadcast Electron Media. (2014) 58:59–75. doi: 10.1080/08838151.2013.875018

[38] Wang J. Youth fitness and physical training in the era of Mobile internet. China Youth Stud. (2019) 12:4–12. doi: 10.19633/j.cnki.11-2579/d.2019.0154

[39] Gilmore JN. Everywear: the quantified self and wearable fitness technologies. New Media Soc. (2016) 18:2524–39. doi: 10.1177/1461444815588768

[40] Lupton D. You are your data: self-tracking practices and concepts of data. In: Selke S (editor) Lifelogging: Digital self-tracking and lifelogging-between disruptive technology and cultural transformation. Wiesbaden: Springer (2016). 61–79.

[41] Sharon T Zandbergen D. From data fetishism to quantifying selves: self-tracking practices and the other values of data. New Media Soc. (2017) 19:1695–709. doi: 10.1177/1461444816636090

[42] Palfrey J Gasser U. Born digital: understanding the first generation of digital natives. New York: Basic Books (2011).

[43] Chen J. An analysis of the status quo,Problems and prospects of "internet plus fitness" from the fitness applications. China Sport Sci. (2016) 36:20–7. doi: 10.16469/j.css.201609003

[44] University of Kentucky Liu D Li X Nicholls State University Santhanam R. Digital games and beyond: what happens when players compete? MIS Q. (2013) 37:111–24. doi: 10.25300/MISQ/2013/37.1.05

[45] Liu CH Wang QM Qian JW. The influence of sports apps on the promotion of physical exercise behavior and the formation of exercise habits. J Nanjing Sport Institute. (2015) 29:109–15. doi: 10.15877/j.cnki.nsic.2015.03.018

[46] Grimes RL. Beginnings in ritual studies. Columbia, S.C: University of South Carolina Press (1995).

[47] Luo H. Thorough Perspective into the Subculture of“Online Prayer”. Humanit Soc Sci J Hainan Univ. (2020) 38:143–9. doi: 10.15886/j.cnki.hnus.2020.02.018

[48] Zhou B. From dependence to solidification:an analysis of the sports sign-inPhenomenon of college students in the new era. J Nanjing Sports Inst. (2020) 19:30–6. doi: 10.15877/j.cnki.nsin.2020.01.006

[49] Zhou Y. A reflective analysis on the popularity of “check-in culture” People's Tribune (2019). 33 p.

[50] Wu Y Knottnerus JD. The formation of ritualized behavior in daily life: from lei Feng’s diary to the diaries of the educated youth. Society. (2007) 27:98–119+208. doi: 10.15992/j.cnki.31-1123/c.2007.01.005

[51] Sun WZ. Task-oriented online check-in: Datafied life and self-management in the new media era. Nanjing J Soc Sci. (2020). 100–107+131. doi: 10.15937/j.cnki.issn1001-8263.2020.06.014

[52] Yan F. “Show culture” in the context of self-media and the new paradigm of contemporary youth self-identity. China Youth Stud. (2015) 6:83–6. doi: 10.19633/j.cnki.11-2579/d.2015.06.014

[53] Zhu L. Self-performance and meaning production: a study on the phenomenon of “internet show-off addiction” among youth groups. Media. (2019) 13:90–3.

[54] Lupton D. The digitally engaged patient: self-monitoring and self-care in the digital health era. Soc Theory Health. (2013) 11:256–70. doi: 10.1057/sth.2013.10

[55] Huang Q Deng X. An analysis of the Mediatization phenomenon in public sports participation. J Wuhan Sports Univ. (2017) 51:25–9. doi: 10.15930/j.cnki.wtxb.2017.11.004

[56] Jia C Tu J. Physical awakening and self-discovery: digital fitness practices of urban full-time housewives in the Mobile internet era. Sports Sci. (2023) 44:42–49+41. doi: 10.13598/j.issn1004-4590.2023.03.014

[57] Qian J. The fleshly spectacle and digital body in communication: an examination from the perspective of media technology. News Writ. (2020) 11:20–7.

